# Prognostic Nutritional Index Is a Predictive Marker for Health-Related Quality of Life in Patients with Adult Degenerative Scoliosis

**DOI:** 10.3390/nu15224771

**Published:** 2023-11-13

**Authors:** Haoran Zhang, You Du, Yiwei Zhao, Yang Yang, Jianguo Zhang, Shengru Wang

**Affiliations:** Department of Orthopedic Surgery, Peking Union Medical College Hospital, Peking Union Medical College and Chinese Academy of Medical Sciences, Beijing 100730, China; zhanghaoran96@126.com (H.Z.); duyou1994@gmail.com (Y.D.); zywspine@163.com (Y.Z.); kaido137@hotmail.com (Y.Y.); jgzhang_pumch@yahoo.com (J.Z.)

**Keywords:** nutritional status, quality of life, adult degenerative scoliosis, spinal surgery

## Abstract

Our aim was to ascertain whether the prognostic nutritional index (PNI), could predict the health-related quality of life (HRQOL) in patients with adult degenerative scoliosis (ADS) undergoing corrective surgery. We conducted a retrospective analysis of consecutive patients diagnosed with ADS between January 2013 and June 2021. Three nutritional parameters were employed for analysis (PNI, anemia, and hypoalbuminemia). We utilized the Scoliosis Research Society-22 (SRS-22) questionnaire and the Oswestry Disability Index (ODI) questionnaire to assess clinical outcomes. Following the epidemiology guidelines, we presented results from three different models: the crude model, minimally adjusted model, and fully adjusted model. A total of 316 ADS patients were included in the statistical analysis. There was no significant difference in sagittal plane radiographic parameters between the two groups. After adjusting for important confounding factors, PNI was an independent predictor of postoperative HRQOL. Specifically, for each one-unit increase in PNI, there was an approximately 20% higher likelihood of patients achieving a better HRQOL. Furthermore, we did not observe an association between hemoglobin levels or albumin levels and HRQOL. In this study, PNI has been demonstrated to be correlated with the postoperative HRQOL in patients with ADS undergoing corrective surgery.

## 1. Introduction

As the population ages, adult degenerative scoliosis (ADS) has plagued more and more people and brought a living and financial burden on patients and their families. ADS is the most common form of adult spinal deformity, which is defined as the spinal deformity in a skeletally mature patient with a scoliotic angle of greater than ten and without a history of adolescent idiopathic scoliosis during childhood and adolescence. According to the literature, the prevalence of ADS ranges from 8.3% to 68% [1,2]. With the advent of aging populations in an increasing number of countries and regions worldwide, the prevalence of ADS is expected to rise further. The primary manifestations of ADS include spinal deformities, neurological deficits, impaired mobility, decreased health-related quality of life (HRQOL), and even lifelong disability. Surgery is the optimal choice in cases where conservative treatment fails or when neurological deficits progress rapidly.

Emerging evidence suggests that perioperative protein malnutrition, particularly baseline nutritional status, is closely associated with the development and outcomes of spinal disorders. Acarbas et al. assessed the impact of the preoperative prognostic nutritional index (PNI) on patients undergoing spinal surgery, revealing that advanced age, lower PNI, and diabetes were significant and independent predictors of perioperative adverse events [3]. PNI, evaluated by the lymphocyte count of the peripheral blood and serum albumin, is a biomarker that integrates nutritional status, immune state, and inflammation condition. The Neurological Surgeons Systematic Review and Evidence-Based Guidelines also noted the correlation between preoperative malnutrition in patients with spinal disorders and a higher incidence of surgical site infections, wound complications, non-union, and readmissions [4]. Furthermore, several studies had independently examined the influence of preoperative nutritional status on medical complications following spinal surgery, including surgical site infections and postoperative delirium [5,6,7,8]. Regrettably, none of these outcome measures encompass HRQOL.

The baseline nutritional status and its relationship with HRQOL have been discussed in several subspecialties within internal medicine and surgery. Cepeda et al. have established the correlation between the HRQOL and the nutritional status of hemodialysis patients [9]. The results indicated a close relationship between HRQOL and nutritional status, emphasizing that maintaining good nutritional status could have a positive impact on HRQOL. A similar trend was observed in the cancer population. A systematic review had demonstrated an association between the prognosis of elderly cancer patients and nutritional status assessed using the mini nutritional assessment, nutritional risk screening, and PNI [10]. The findings indicated that poor nutritional status was linked to lower survival rates, reduced treatment completion rates, and higher healthcare costs.

In this retrospective cohort study, our aim was to ascertain whether the PNI, as a non-invasive nutritional status biomarker, could predict the HRQOL in patients with ADS undergoing corrective surgery. Considering that anemia and hypoalbuminemia were clinically easily available and commonly used indicators related to nutritional status, we also further explored whether anemia and hypoalbuminemia were associated with the HRQOL.

## 2. Methods

### 2.1. Patient Selection

We conducted a retrospective analysis of consecutive patients diagnosed with ADS who underwent corrective surgery between January 2013 and June 2021. Inclusion criteria encompassed the following: (1) patients aged 45 years or older; (2) a coronal Cobb angle exceeding 10°; (3) availability of complete radiographic data and clinical outcome measurements; and (4) a minimum follow-up period of 2 years. Exclusion criteria were defined as follows: (1) a prior diagnosis of scoliosis or other spinal deformities; (2) a history of prior spinal surgery; (3) a history of spinal trauma, spinal tumor, ankylosing spondylitis, or spinal tuberculosis. Ultimately, our study incorporated a total of 316 ADS patients who met these criteria. This study adhered to the principles of the Declaration of Helsinki and received approval from the Institutional Review Board of our institution. All participants provided informed consent prior to undergoing surgery.

### 2.2. Data Collection

The data were obtained retrospectively from the medical record system. Demographic data collected included age, sex, body mass index (BMI), age adjusted Charlson comorbidity index (aCCI), and osteopenia. Perioperative data collected included length of hospital stay, operative duration, estimated blood loss, American Society of Anesthesiologists (ASA) data, number of fusion levels, and osteotomy (with or without three-column osteotomy). The ASA was a system to assess a patient’s physical health status and clinical risk during anesthetic administration and surgical operation. Preoperatively, the patient is subjectively assigned a score according to their physical status, which is determined by the anesthesiologist after considering patient presentation, history, and functional limitations. Assigning this score—ranked ASA 1 through ASA 6—would thereby attempt to categorize the patient’s risk of perioperative complications based on their physical status and overall health.

Radiographic measurements were obtained at baseline and at the last follow-up (at least 2 years postoperatively). In standard lateral X-rays of the whole spine, the measurements were pelvic tilt (PT), lumbar lordosis (LL), pelvic incidence minus lumbar lordosis mismatch (PI-LL), and sagittal vertical axis (SVA). LL was defined as the angle between the superior endplate of T12 and S1. PI-LL was calculated using the following formula: pelvic incidence minus LL. SVA was defined as the horizontal distance between the posterosuperior corner of the sacrum and the C7 plumb line. All radiographs were assessed using software version 2.3.2.1 (Surgimap, Nemaris Inc., New York, NY, USA). All imaging indicators were independently measured by two researchers and then averaged for statistical analysis.

We employed three nutritional status indicators for analysis. Nutritional indicators were collected and measured on admission. The PNI value was calculated as serum albumin (g/L) + 5 × lymphocyte count (10^9^/L). According to the classification criteria from previous literature, the overall population was divided into two groups: PNI < 50 and PNI ≥ 50 [8]. Anemia was characterized as hemoglobin levels below 130 g/L for males and below 120 g/L for females. Hypoalbuminemia was established when the serum albumin level was less than 35 g/L.

### 2.3. Clinical Outcomes

We utilized the Scoliosis Research Society-22 (SRS-22) questionnaire and the Oswestry Disability Index (ODI) score to assess clinical outcomes. These questionnaires were collected at the last follow-up visit of the patients. These questionnaires were considered effective measures for evaluating spinal deformities. Evidence suggests that the SRS-22 and ODI are the only patient-reported outcome measures that have undergone rigorous evaluation for their measurement properties within the adult scoliosis population [11]. The SRS-22questionnaire is a simple and practical disease-specific patient-based measure of treatment effectiveness for patients with spinal deformity, and has been a widely used instrument. The SRS-22 questionnaire, which measures five domains: pain, activity, appearance, mental, and satisfaction, has already been proven to be reliable, valid, and responsive to change. The ODI was first designed in 1980 to assess low back pain disability and was subsequently revised in 2000. It has been widely used and validated for thoracic and lumbar spine pain [11]. In our study, the favorable clinical outcomes were defined as a total SRS-22 score ≥ 4 or a total ODI score ≤ 20 at the last follow-up [12,13].

### 2.4. Statistical Analysis

The study data were presented in the following manner: continuous variables were expressed as the mean ± standard deviation, while categorical variables were represented as proportions. Statistical comparisons were made using Student’s *t*-test, analysis of variance, or Chi-square test as appropriate. To investigate the statistical association between nutritional status and clinical outcomes, logistic regression models were employed. Following the guidelines set forth by the Strengthening the Reporting of Observational Studies in Epidemiology statement [14], we presented results from three different models: the crude model, minimally adjusted model, and fully adjusted model. Our criteria for selecting covariates to include in the multivariate model were as follows: firstly, if the introduction of a covariate into the base model or removal it from the full model resulted in a change of more than 10% in the regression coefficient of the independent variable, this covariate was considered for inclusion [15]. Additionally, variables with a *p* value of less than 0.10 in the univariate analysis were included. Finally, we incorporated appropriate covariates based on existing literature and clinical expertise at our institution. Covariates included sex, age, BMI, aCCI, osteopenia, ASA, PT, PI-LL, LL, and SVA.

A two-tailed *p* < 0.05 was considered statistically significant. All statistical analyses were conducted using the Statistical Package for Social Sciences version 27.0 software (SPSS, Inc., Chicago, IL, USA) and R version 4.3.1 for Windows (R Foundation for Statistical Computing, Vienna, Austria).

## 3. Results

### 3.1. Demographic Data

After screening based on inclusion and exclusion criteria, a total of 316 ADS patients who underwent spinal corrective surgery were included in the subsequent statistical analysis. The entire cohort was divided into two groups according to baseline nutritional status: PNI < 50 (poor nutritional status) and PNI ≥ 50 (satisfactory nutritional status). Patients with poor nutritional status tended to be older (67.2 years vs. 64.8 years), but this difference was not statistically significant. Gender distribution was approximately equal in both groups. Patients with poor nutritional status generally had lower BMI (26.0 kg/m^2^ vs. 27.6 kg/m^2^) and a higher burden of comorbidities (5.0 vs. 4.5). Bone quality conditions were largely consistent between the two groups (Table 1).

### 3.2. Perioperative Data

Patients with poor nutritional status had significantly longer hospital stays compared to those with satisfactory nutritional status (11.4 days vs. 10.3 days). Additionally, their general condition was poor (ASA score 2.3 vs. ASA score 2.1). Intraoperative estimated blood loss and operative duration were similar between the two groups. Regarding surgical details, the proportion of the number of fusion levels and three-column osteotomy was comparable in both groups (Table 1).

### 3.3. Radiographic Measurements

Four sagittal plane radiographic parameters were compared both preoperatively and during follow-up. In terms of PT, the data for both groups during follow-up were lower than their preoperative values (29.2° vs. 25.9°; 28.0° vs. 25.8°), indicating a reduction in the degree of pelvic rotation compensation. However, there was no significant difference in PT between the two groups at follow-up. Regarding PI-LL, both groups’ follow-up data were lower than their preoperative data, with no significant difference between the two groups. This suggested further alignment of spinal pelvic parameters. Similarly, LL showed significant improvement, with the average preoperative LL being 9.6° and the average LL during postoperative follow-up being 23.4°. Regarding overall sagittal plane balance, both groups demonstrated noticeable improvements (SVA 4.0 cm vs. 5.8 cm; SVA 3.9 cm vs. 5.6 cm), with no significant difference between the two groups (Table 1).

### 3.4. Nutritional Status

Patients with PNI < 50 had an average PNI of 45.8, while patients with PNI ≥ 50 had an average PNI of 52.7, and this difference was statistically significant (*p* < 0.001). Additionally, patients with PNI < 50 were more frequently associated with hypoalbuminemia (16.7%), whereas the occurrence rate in those with PNI ≥ 50 was lower (9.2%), and this difference was statistically significant. Approximately 20% of patients suffered from anemia, and the proportion of anemic patients was similar in both groups (21.7% vs. 19.9%).

### 3.5. PNI and SRS-22

The association between PNI and SRS-22 score was assessed in the multivariate model, with confounding factors identified as sex, age, BMI, aCCI, osteopenia, ASA, PT, PI–LL, LL, and SVA. When PNI was included in the model as a continuous variable, it was found that for every increase of one unit in PNI, the odds of patients achieving a satisfactory SRS score increased by 23% (OR = 1.231, 95%CI 1.153–1.315, *p* < 0.001), after adjusting for other confounding factors. Furthermore, when PNI was introduced into the model as a categorical variable, it was observed that patients with PNI ≥ 50 had 2.86 times higher odds of achieving a satisfactory SRS score compared to patients with PNI < 50 (OR = 2.867, 95%CI 1.708–4.812, *p* < 0.001) (Table 2).

### 3.6. PNI and ODI

The association between PNI and ODI score was assessed in the multivariate model, with confounding factors identified as sex, age, BMI, aCCI, ASA, PT, PI–LL, and SVA. When PNI was included in the model as a continuous variable, it was found that for every increase of one unit in PNI, the odds of patients achieving a satisfactory ODI score increased by 18% (OR = 1.175, 95% CI 1.107–1.247, *p* < 0.001), after adjusting for other confounding factors. Furthermore, when PNI was introduced into the model as a categorical variable, it was observed that patients with PNI ≥ 50 had 2.08 times higher odds of achieving a satisfactory ODI score compared to patients with PNI < 50 (OR = 2.088, 95% CI 1.307–3.336, *p* = 0.002) (Table 3).

### 3.7. Anemia and Hypoalbuminemia

The potential impacts of anemia and hypoalbuminemia on HRQOL were also evaluated. The results of the multivariate regression model showed that neither anemia nor hypoalbuminemia was statistically significantly associated with postoperative HRQOL in ADS patients. Although hypoalbuminemia exhibited a marginally significant association with SRS-22 and ODI in the crude model, this relationship ceased to be significant in the fully adjusted model (*p* = 0.125; *p* = 0.414) (Table 4).

## 4. Discussion

With the aging of the population, ADS is set to become more prevalent. Therefore, it is imperative to continue developing and improving tools for the prevention, screening, and treatment of patients with ADS. Given the advanced age and multiple comorbidities often seen in these patients, they frequently exhibit poor nutritional status. Additionally, the nutritional status of ADS patients scheduled for surgery may deteriorate due to psychological stress, loss of appetite, mobility impairments caused by gastroesophageal reflux disease, and severe pain [16]. Malnourished patients are not only more prone to various complications during surgery but also face suboptimal long-term outcomes [17,18].

To the best of our knowledge, this is the first investigation into whether baseline nutritional status is correlated with postoperative HRQOL in patients with ADS. In this study, after adjusting for important confounding factors identified in the literature and statistics, PNI was an independent predictor of postoperative HRQOL. Specifically, for each 1-unit increase in PNI, there was an approximately 20% higher likelihood of patients achieving a satisfactory HRQOL. Furthermore, we did not observe an association between hemoglobin levels or albumin levels and HRQOL, despite these two indicators being commonly used by clinicians to assess nutritional status.

The PNI is assessed through a combination of serum albumin levels and peripheral blood lymphocyte counts, making it a comprehensive biomarker that reflects nutritional status, immune function, and inflammatory conditions. This index was developed by Buzby and colleagues in 1980 and was initially used to evaluate perioperative nutritional status in gastrointestinal surgery [19]. In recent years, PNI has gradually become a marker for predicting outcomes in various diseases, including kidney disease [20], gastric cancer [21], and cardiomyopathy [22]. In the fields of spinal surgery and neurosurgery, PNI has also been employed to predict clinical outcomes such as medical complications [8], postoperative psychological disorders [7], and perioperative adverse events [3]. Current research demonstrates that PNI is equally effective in predicting postoperative HRQOL.

Addressing the unchangeable risk factor before surgery may be challenging, but it is important to note that interventions to address malnutrition are not only feasible but also recommended. In fact, the European Society for Clinical Nutrition and Metabolism guidelines advocate for nutritional therapy in the 7–10 days leading up to surgery if at least one of the following criteria applies: (1) if a patient has experienced weight loss exceeding 10–15% within the last 6 months; (2) when BMI falls below 18.5 kg/m^2^; (3) if the patient is graded as subjective global assessment grade C; (4) when the nutritional risk screening score exceeds 5; and (5) if the preoperative serum albumin level is less than 3 g/L (provided there is no evidence of hepatic or renal failure) [23]. These guidelines emphasize the importance of identifying and addressing malnutrition in patients before surgery, as it can have a significant impact on surgical outcomes and overall patient well-being.

For cases of severe malnutrition, in addition to the measures mentioned above, long-term nutritional interventions and exercise therapy may be necessary [24]. Based on these guidelines and recommendations, for patients with ADS planning to undergo corrective surgery, comprehensive nutritional assessments should be conducted preoperatively, and nutritional interventions and exercise therapy should be considered as needed. These interventions not only reduce the risk of short-term medical complications and reoperations but also promote long-term improvements in HRQOL, ultimately helping patients reintegrate into society. The exercise therapy consisted of a combination of strength, flexibility, balance, and endurance training. At the outset, the trained case managers evaluated the physical fitness of each participant based on multiple facets (handgrip strength, gait speed, upper and lower body flexibility, lower extremity strength, balance and leg strength, and volume of physical activity). In order to enhance participants’ fitness levels, the participants received personalized (fitted to individual needs) exercise prescriptions and handy tools (e.g., resistance band, grip-ball, and pedometer) from the licensed physiotherapists. Approximately three to seven exercise sessions per week were recommended, with the time (5 to 60 min) per session or repetitions tailored to participants’ capabilities.

Oe and colleagues conducted a prospective nutritional intervention study on malnourished patients scheduled for adult spinal deformity surgery to investigate the incidence of postoperative complications and the natural nutritional status history [5]. For patients with a PNI less than 50, the institution’s nutritionists provided nutritional guidance, instructing them to consume nutritional supplement beverages three times a day (providing 200 kcal of energy, 7.5 g of protein, 5.6 g of lipid, and 31.7 g of carbohydrates). Nutritionists assessed their nutritional status and provided recommendations on a monthly basis. The results indicated that nutritional intervention might reduce the deterioration of nutritional status and postoperative complications. However, the authors noted that the effects of nutritional intervention alone may be limited, and prehabilitation with exercise therapy might be necessary. Gillis and colleagues also reported that a key aspect of prehabilitation is combining exercise therapy with nutritional therapy, with exercise therapy requiring 4 to 5 weeks to be effective [25].

This study has several strengths. Firstly, it includes one of the largest cohorts of patients with ADS undergoing surgery. This sample size allows for the detection of statistically significant associations that might be challenging to uncover in smaller studies, and it helps reduce the risk of false negatives to a certain extent. Secondly, nutritional status was assessed using the PNI, a well-established and objectively measured indicator that can be obtained through routine preoperative laboratory tests, ensuring fairness in the assessment. Finally, this research specifically targeted patients with ADS, aiming to assess the impact of preoperative nutritional status while controlling for the influence of relevant confounding factors. This pure cohort design ensures that the conclusions are more generalizable to ADS, compared to cohorts that include adult spinal deformity without distinguishing between adult idiopathic scoliosis and ADS.

Certainly, our study has some limitations. Firstly, this study is a retrospective cohort study, and it is important to acknowledge that there may be challenges in entirely avoiding recall bias and selection bias. To minimize the potential impacts of bias, we meticulously established and applied stringent inclusion and exclusion criteria. These criteria were designed to enhance the robustness of our study and minimize the likelihood of bias to the greatest extent possible. Secondly, this research was conducted at a single center, focusing on Chinese patients undergoing spinal deformity surgery. Therefore, the generalizability of our findings to other ethnic groups may be limited. Thirdly, the study population was restricted to patients with ADS, so whether the corresponding conclusions are applicable to adult idiopathic scoliosis or congenital scoliosis requires further confirmation.

## 5. Conclusions

In this retrospective cohort study, PNI has been demonstrated to be correlated with the postoperative HRQOL in patients with ADS undergoing corrective surgery. This work underscores the need for future research to investigate interventions that can improve the perioperative nutritional status of these patients. Furthermore, studies similar to ours should be conducted in other types of spinal deformities, as this would help determine which patients stand to benefit the most from nutritional interventions.

## Figures and Tables

**Table 1:** Clinical features of the patients stratified across the PNI. Values were expressed as number (%) or mean ± SD.

Variables	Overall (N = 316)	PNI < 50 (N = 120)	PNI ≥ 50 (N = 196)	*p* Value
Age, years	65.7 ± 11.9	67.2 ± 12.1	64.8 ± 11.7	0.079
Sex				0.901
Female	212 (67.1%)	80 (66.7%)	132 (67.3%)	
Male	104 (32.9%)	40 (33.3%)	64 (32.7%)	
BMI, kg/m^2^	27.0 ± 5.9	26.0 ± 5.7	27.6 ± 6.1	0.021
aCCI	4.6 ± 1.7	5.0 ± 1.6	4.5 ± 1.8	0.012
Osteopenia				0.897
Yes	125 (50.0%)	49 (50.5%)	76 (49.7%)	
No	125 (50.0%)	48 (49.5%)	77 (50.3%)	
Length of stay, days	10.7 ± 4.1	11.4 ± 4.1	10.3 ± 4.0	0.021
Estimated blood loss, ml	654.6 ± 444.3	665.2 ± 391.1	648.2 ± 474.8	0.741
Operative duration, min	281.9 ± 68.1	286.2 ± 60.2	279.2 ± 72.6	0.375
ASA score	2.2 ± 0.7	2.3 ± 0.7	2.1 ± 0.7	0.024
Number of fusion levels	4.9 ± 2.1	4.9 ± 2.1	4.9 ± 2.1	0.804
Three-column osteotomy				0.763
Yes	82 (25.9%)	30 (25.0%)	52 (26.5%)	
No	234 (74.1%)	90 (75.0%)	144 (73.5%)	
Preoperative PT (°)	28.4 ± 9.8	29.2 ± 8.9	28.0 ± 10.4	0.284
PT at follow up (°)	25.8 ± 10.6	25.9 ± 10.8	25.8 ± 10.5	0.947
Preoperative PI-LL (°)	34.5 ± 12.6	34.9 ± 12.8	34.2 ± 12.5	0.647
PI-LL at follow up (°)	20.7 ± 8.5	21.4 ± 7.7	20.2 ± 8.9	0.217
Preoperative LL (°)	9.6 ± 8.5	9.4 ± 6.1	9.7 ± 5.7	0.716
LL at follow up (°)	23.4 ± 14.1	22.9 ± 13.1	23.7 ± 14.3	0.621
Preoperative SVA (cm)	5.7 ± 3.8	5.8 ± 3.6	5.6 ± 3.9	0.635
SVA at follow up (cm)	4.0 ± 2.1	4.0 ± 2.0	3.9 ± 2.2	0.744
SRS-22 score at follow up	3.7 ± 0.9	3.4 ± 1.0	3.9 ± 0.7	<0.001
ODI at follow up	18.1 ± 5.5	19.3 ± 6.7	17.4 ± 4.4	0.002
PNI	50.1 ± 3.7	45.8 ± 2.0	52.7 ± 1.3	<0.001
Anemia				0.706
Yes	65 (20.6%)	26 (21.7%)	39 (19.9%)	
No	251 (79.4%)	94 (78.3%)	157 (80.1%)	
Hypoalbuminemia				0.047
Yes	38 (12.0%)	20 (16.7%)	18 (9.2%)	
No	278 (88.0%)	100 (83.3%)	178 (90.8%)	

PNI, prognostic nutritional index; BMI, body mass index; aCCI, age-adjusted Charlson comorbidity index; ASA, American Society of Anesthesiologists; PT, pelvic tilt; PI-LL, pelvic incidence minus lumbar lordosis mismatch; LL, lumbar lordosis; SVA, sagittal vertical axis; SRS-22, scoliosis research society-22; ODI, Oswestry disability index.

**Table 2:** Unadjusted and adjusted model for favorable SRS-22 score. Values are odds ratio (OR) (95% CI).

Variable	Crude Model ^a^	Minimally Adjusted Model ^b^	Fully Adjusted Model ^c^
Continuous PNI	1.357 (1.252–1.470) *p* < 0.001	1.299 (1.195–1.411) *p* < 0.001	1.231 (1.153–1.315) *p* < 0.001
Categorical PNI			
<50	Reference	Reference	Reference
≥50	4.188 (2.479–7.075) *p* < 0.001	3.359 (2.070–5.451) *p* < 0.001	2.867 (1.708–4.812) *p* < 0.001

^a^ Crude model: we did not adjust other covariants. ^b^ Minimally adjusted model: we adjusted sex, age, BMI, aCCI, and ASA. ^c^ Fully adjusted model: we adjusted sex, age, BMI, aCCI, osteopenia, ASA, PT, PI–LL, LL, and SVA. PNI, prognostic nutritional index; BMI, body mass index; aCCI, age-adjusted Charlson comorbidity index; ASA, American Society of Anesthesiologists; PT, pelvic tilt; PI-LL, pelvic incidence minus lumbar lordosis mismatch; LL, lumbar lordosis; SVA, sagittal vertical axis; SRS-22, Scoliosis Research Society-22.

**Table 3:** Unadjusted and adjusted model for favorable ODI score. Values are odds ratios (OR) (95% CI).

Variable	Crude Model ^a^	Minimally Adjusted Model ^b^	Fully Adjusted Model ^c^
Continuous PNI	1.302 (1.198–1.416) *p* < 0.001	1.210 (1.134–1.291) *p* < 0.001	1.175 (1.107–1.247) *p* < 0.001
Categorical PNI			
<50	Reference	Reference	Reference
≥50	3.121 (1.930–5.049) *p* < 0.001	2.490 (1.498–4.139) *p* < 0.001	2.088 (1.307–3.336) *p* = 0.002

^a^ Crude model: we did not adjust other covariants. ^b^ Minimally adjusted model: we adjusted sex, age, BMI, aCCI, and ASA. ^c^ Fully adjusted model: we adjusted sex, age, BMI, aCCI, ASA, PT, PI–LL, and SVA. PNI, prognostic nutritional index; BMI, body mass index; aCCI, age-adjusted Charlson comorbidity index; ASA, American Society of Anesthesiologists; PT, pelvic tilt; PI-LL, pelvic incidence minus lumbar lordosis mismatch; SVA, sagittal vertical axis; ODI, Oswestry disability index.

**Table 4:** Relationship between anemia and hypoalbuminemia and clinical outcomes. Values are odds ratios (OR) (95% CI).

Variable	Crude Model ^a^	Minimally Adjusted Model ^b^	Fully Adjusted Model ^c^
SRS-22 score			
Anemia			
Yes	Reference	Reference	Reference
No	1.331 (0.621–2.849) *p* = 0.462	1.247 (0.621–2.502) *p* = 0.534	1.220 (0.607–2.452) *p* = 0.577
Hypoalbuminemia			
Yes	Reference	Reference	Reference
No	1.676 (0.911–3.085) *p* = 0.097	1.563 (0.858–2.850) *p* = 0.145	1.492 (0.895–2.487) *p* = 0.125
ODI score			
Anemia			
Yes	Reference	Reference	Reference
No	1.328 (0.620–2.845) *p* = 0.465	1.185 (0.445–3.157) *p* = 0.734	1.085 (0.416–2.830) *p* = 0.867
Hypoalbuminemia			
Yes	Reference	Reference	Reference
No	1.553 (0.957–2.520) *p* = 0.075	1.401 (0.667–2.943) *p* = 0.374	1.378 (0.638–2.977) *p* = 0.414

^a^ Crude model: we did not adjust other covariants. ^b^ Minimally adjusted model: we adjusted sex, age, BMI, aCCI, and ASA. ^c^ Fully adjusted model: we adjusted sex, age, BMI, aCCI, ASA, PT, PI–LL, and SVA. BMI, body mass index; aCCI, age-adjusted Charlson comorbidity index; ASA, American Society of Anesthesiologists; PT, pelvic tilt; PI-LL, pelvic incidence minus lumbar lordosis mismatch; SVA, sagittal vertical axis; SRS-22, Scoliosis Research Society-22; ODI, Oswestry disability index.

## Data Availability

The datasets used and/or analyzed during the current study are available from the corresponding author on reasonable request.
